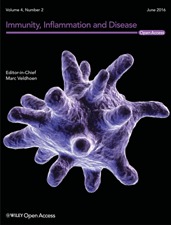# Issue Information

**DOI:** 10.1002/iid3.63

**Published:** 2016-05-25

## Abstract